# May Failure to Thrive in Infants Be a Clinical Marker for the Early Diagnosis of Cow’s Milk Allergy?

**DOI:** 10.3390/nu12020466

**Published:** 2020-02-13

**Authors:** Lucia Diaferio, Davide Caimmi, Maria Carmen Verga, Valentina Palladino, Lorenzo Trovè, Paola Giordano, Elvira Verduci, Vito Leonardo Miniello

**Affiliations:** 1 Department of Pediatrics, Giovanni XXIII Hospital, University of Bari, 70126 Bari, Italy; valentinapalladino@hotmail.it (V.P.); lorenzo.trove@yahoo.com (L.T.); paola.giordano@uniba.it (P.G.); vito.miniello@libero.it (V.L.M.); 2 Département de Pneumologie et Addictologie, Allergy Unit, Hôpital Arnaud de Villeneuve, CHU de Montpellier, Univ Montpellier, 34000 Montpellier, France; davide.caimmi@gmail.com; 3 Epidemiology of Allergic and Respiratory Diseases Department (EPAR), IPLESP, UMR 1136 INSERM—Sorbonne Université, 75571 Paris, France; 4 Primary Care Pediatrics, ASL Salerno, 84019 Vietri sul Mare (SA), Italy; vergasa@virgilio.it; 5 Department of Pediatrics, San Paolo Hospital, Department of Health Science, University of Milan, 20142 Milan, Italy; elvira.verduci@unimi.it

**Keywords:** food allergy, failure to thrive, non-IgE-mediated food allergy, cow’s milk allergy

## Abstract

Objectives—Failure to thrive (FTT) in infants is characterized by growth failure. Although, cow’s milk allergy (CMA) may have an impact on growth and leads to FTT, data are still limited. We focused on FTT as a possible clinical marker for an early diagnosis of CMA. The aim of the present study was to evaluate the implications of cow’s milk hypersensitivity in infants with FTT and the growth catch-up after a cow’s milk-free diet (CMFD). Methods—A cross-sectional study of all consecutive infants evaluated at the Pediatric Nutrition and Allergy Unit of the University Hospital of Bari (Italy) from January 2016 to April 2018 with a medical-driven diagnosis of FTT. Eligible infants were investigated for possible IgE mediated or non-IgE mediated CMA. Results—43 infants were included, mean age 5.7 months. 33/43 (77%) FTT presented a CMA related disease: 3/43 (7%) were diagnosed as presenting an IgE mediated CMA, 30 (93%) had a non IgE-mediated CMA, confirmed by the elimination diet for diagnostic purposes, that led to a significant improvement of symptoms and recrudescence after milk reintroduction. A total of 29 out of 30 patients (one patient was lost at follow-up) moved up to their original growth percentile after dietary changes. Growth z-scores were computed based on WHO anthropometric data. In 10 out of 43 patients (23%) were diagnosed with gastro-esophageal reflux disease (GERD). Conclusions—when evaluating an infant with FTT, physicians should include in their evaluation an extensive search for IgE mediated and non IgE mediated CMA. When in vivo and in vitro analysis are not conclusive, a 4- to 8-weeks trial of CMFD and a consecutive re-introduction of milk proteins may be helpful in less common diagnoses.

## 1. Introduction

The term failure to thrive (FTT) is used to indicate an inadequate growth in early childhood, and is characterized by growth failure [1]. It may be determined by several different etiologies, through different underlying mechanisms, such as: Decreased calorie intake, inadequate caloric absorption, and normal calorie intake with an increased metabolic need [1]. FTT may be defined as a decelerated or arrested physical growth (i.e., weight-for-age and/or weight-for-length measurements fall below the third or the fifth percentile on more than one consecutive assessment, or weight deceleration crosses two major percentile lines on age-appropriate growth centile charts). When FTT arises from nutritional insufficiency, weight usually declines from the baseline percentile before length does. Currently, FTT is classified as an illness-related or a non–illness-related condition [2]. It has also been suggested that FTT could be a multifactorial condition, possibly associated with food-induced gastrointestinal allergic disorders. 

In infants, cows’ milk allergy (CMA) is the most common food allergy (FA) [3]. Data show that both IgE and non-IgE mediated FA prevalence is increasing in western countries [4,5,6].

FA diagnosis is based on patient’s reported clinical history, clinical features, and physical examination and, in case of symptoms suggesting an IgE mediated mechanism, the results of in vivo and in vitro tests [7]. 

As for CMA, the diagnosis is particularly challenging due to a wide range of presenting symptoms, including acute (cutaneous, gastrointestinal (GI), respiratory, or even anaphylactic), delayed (mainly gastrointestinal), or chronic manifestations, especially associated with non-IgE-mediated CMA often leading to a misdiagnosis or to a diagnostic delay [8].

Among all chronic manifestations, FTT in infants may be considered a clinical marker for an early diagnosis of CMA. 

Oral food challenge (OFC) is still considered as the gold-standard for confirming both the diagnosis of CMA and the possible acquisition of tolerance [9,10,11]. Nevertheless, in infants presenting with non-IgE-mediated symptoms, the oral provocation test requires a prolonged follow-up because of possible delayed reactions, making its interpretation more difficult. For such reason, in clinical setting, a cow’s milk-free diet (CMFD) is often helpful to reach diagnosis, especially in infants and young children, presenting with non-immediate symptoms [9,10,11].

The lack of optimal diagnostic tests and of biomarkers, associated with the presence of diverse, non-specific symptoms, makes it difficult to promptly diagnose infants with non-IgE-mediated CMA [10]. In infants with a diagnosis of FTT, the disappearance of symptoms after a CMFD, and the improvement of linear growth after a dietary intervention, may be the keystone for the diagnosis of CMA, when in vivo and in vitro assessments are not conclusive.

For these reasons, whenever evaluating infants with FTT, clinicians should consider an underlying CMA, especially in those with non-IgE mediated forms.

In September 2014, a panel of experts proposed the use of a Cow’s milk-related symptom score (CoMiSS™) to predict CMA. Such score is based on the presence of different unspecific symptoms, including crying, regurgitation, altered stools (Bristol, UK), skin symptoms, and respiratory symptoms [12]. Nevertheless, the score does not include FTT as a possible symptom evocative of CMA.

The primary objective of the present study was to evaluate, in infants presenting with FTT, the involvement of CMA, and to assess whether the clinical condition could be considered a marker of milk hypersensitivity. The secondary objective involves the evaluation of the CMFD on growth parameters over a period of three months.

## 2. Materials and Methods

### 2.1. Study Design

We conducted a study evaluating data from all consecutive patients with a physician’s diagnosis of FTT and admitted to our Nutrition and Allergy Unit (Department of Pediatrics, “Giovanni XXIII” Hospital, Bari, Italy), between January 2016 and April 2018. Eligibility criteria included: Age ≤ 15 months, birth weight > 2800 g and <4000 g, full-term newborn (37–42 weeks of gestation), single birth, infants having white parents and residing in the Bari region (<50 km). Exclusion criteria were: (a) Antenatal/perinatal problems (i.e., intrauterine infections, inborn errors of metabolism), (b) congenital syndromes (i.e., Down, Silver-Russell), (c) insufficient intake of calories/nutrients (inadequate provision, suck-swallow problems), (d) malabsorption (i.e., celiac disease), (e) chronic medical conditions (i.e., congenital heart disease, genetic disorders, immunodeficiency), (f) neurologic impairment and (g) anatomical malformations (e.g.,: hypertrophic pyloric stenosis), (h) history of repeated acute infections. 

All eligible infants underwent anthropometry and an allergy work-up as described below. 

This study was performed in accordance with the Declaration of Helsinki, and it was independent in all stages of the design and conduction, and for the collection, analysis or interpretation of the data, drafting, review and approval of the paper.

### 2.2. Collected Data

Detailed prenatal and perinatal history, including birth weight and pregnancy complications, were essential to identifying the underlying familiar, metabolic, or endocrine disorders. Anthropometric measures were assessed by trained medical professionals, based on standard procedures. Weight was measured on an electronic digital balance, the infant being fully naked, without nappy/diaper; the same instrument was used for each included patient. Length (to nearest 0.1 cm) was measured on a length board for infants and toddlers. Body mass index (BMI kg/m^2^) was calculated for each infant. We evaluated changes in weight-for-age z-score (WAZ), weight-for-length (WLZ), length-for age (LAZ), BMI-for-age (BMIAZ) and head circumference-for-age (HCA). All these data were then plotted on a WHO 2006 Child Growth Standard scale [13]. Percentiles and *z*-scores were electronically computed by using the WHO Anthro software (www.who.int/childgrowth/software/en/). Children were diagnosed with FTT where they met 1 or more of the standard diagnostic criteria [14].

In case of recurrent vomiting, we also performed radiologic investigations to exclude possible malformations, such as hypertrophic pyloric stenosis.

We also assessed for each patient, their diet history, including feeding schedule and techniques for formula preparation, and we focused on caloric intake of complementary food. Data on feeding were extracted from questionnaires completed by the infant’s parents. Both parents of each patient (or their legal guardians) signed an informed consent to perform the OFC, when needed, and signed an agreement for possible data exploitation. All patients were included in our database, their data was protected, anonymized, and authorized for exploitation, as previously validated by the local ethics committee. Considering the study as part of a clinical routine practice, no additional authorization was needed at that time, following national legislation.

To exclude a diagnosis of IgE mediated CMA, we performed skin prick tests (SPT), both with a standardized extract (Stallergenes-Greer, Milan, Italy), and with pasteurized milk in all patients. Serum-specific IgE to CM were assessed as well (ImmunoCAP^®^ Phadia, Thermo Fisher Scientific, Uppsala, Sweden). In case of positive SPT and/or specific IgE, patients underwent an OFC, which was performed according to EAACI guidelines [15]. 

When a non-IgE gastrointestinal food allergy (non-IgE-GI-FA), such as food protein-induced enteropathy (FPE) or refractory gastro-esophageal reflux disease (GERD) was suspected to be associated with FTT, avoidance of the milk’s proteins was recommended: Extensive hydrolyzed formula (eHF) was prescribed in non-breastfed infants, while CM avoidance in maternal diet was prescribed in breast-fed infants [11]. For each infant who needed such a therapeutic approach, we recommended an eHF respecting the American Academy of Pediatrics hypoallergenicity criteria [16].

Allergen avoidance was maintained for 4 to 8 weeks before a possible reintroduction, as the elimination-reintroduction sequence currently represents the only recommended diagnostic approach for gastrointestinal manifestations of non-IgE-GI-FA in infants [9,10,11].

Food protein-induced allergic proctocolitis (FPIAP) diagnosis was based on a convincing history of consumption of the offending food(s), with the exclusion of other possible diagnoses, and/or a positive allergy work-up. Clinical symptoms suggestive of GERD, length of symptoms, type and duration of GERD dietetic treatment, adherence to treatment, and associated comorbid states were evaluated as well.

All data were recorded in an electronic case report form. Weight-for-age (WFA), length-for-age (LFA), weight-for-length (WFL), body mass index (BMI)-for-age and head circumference-for-age (HCA) z-scores were computed, based on WHO anthropometric data [13]. 

### 2.3. Statistical Analysis

Statistical analyses were performed using STATA version 13 (StataCorp. 2013. *Stata Statistical Software: Release 13*. College Station, TX, USA: StataCorp LP). Continuous variables were expressed as median and standard deviation (SD). Categorical variables were expressed in percentages (%). Differences in subgroups were evaluated through the chi-squared tests. Logistic regression was assessed to evaluate possible association between categorical variables. Odds Ratio (OR) was calculated for the different variables, and we used a 95% interval of confidence. All *p*-values < 0.05 were considered as statistically significant. 

## 3. Results

**Figure 1:**
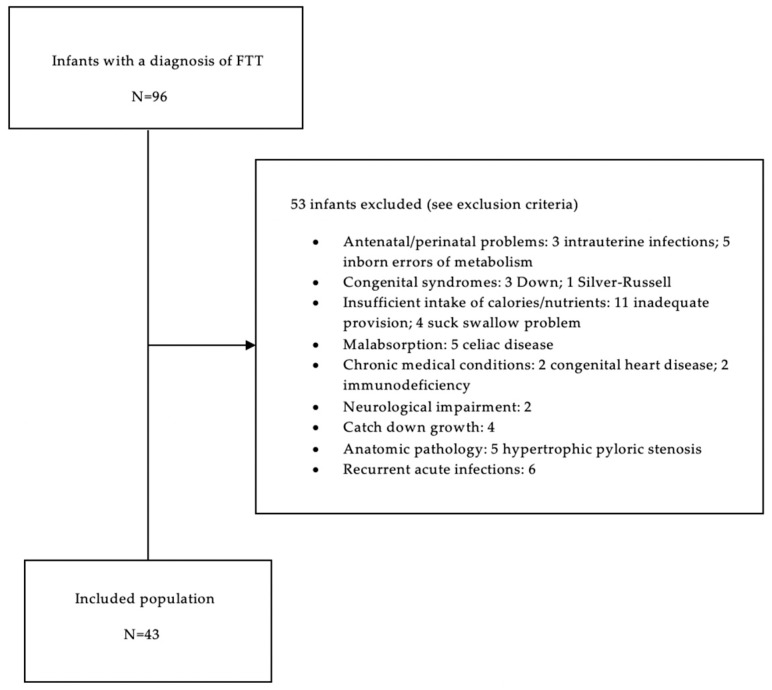
Study flow chart showing the screened cohort of 96 infants, and the included group of 43.

We screened 96 infants with a physician-based diagnosis of FTT. Fifty-three of them were excluded based on our inclusion/exclusion criteria. Therefore, we included in the present study 43 infants with FTT (Figure 1).

Clinical features, diagnosis, and allergic evaluation of the 43 infants are shown in Table 1. 

Most of the patients were females (58%), with mean age of 5.7 months (SD 2.1). They were born with a standard weight, at an average gestational age of 38.7 weeks (SD 1.0) and all of them reported an APGAR score, strictly superior to 7/10 at 1 min after birth. Sixteen (37%) were born though caesarean-section delivery. Between birth and the first evaluation, the mean (±SD) WFA and LFA z-scores was significantly decreased by an average of 0.6 (SD1.0), and 0.5 (SD 1.1), respectively (*p* < 0.001; *p* = 0.003, Student’s test).

A family history of food allergy was reported in 21/43 (49%) infants. In all these 21 patients, a CMFD improved the clinical symptoms, resolving the FTT. The socio-economic status (SES) of included patients is shown in Table 2.

**Figure 2:**
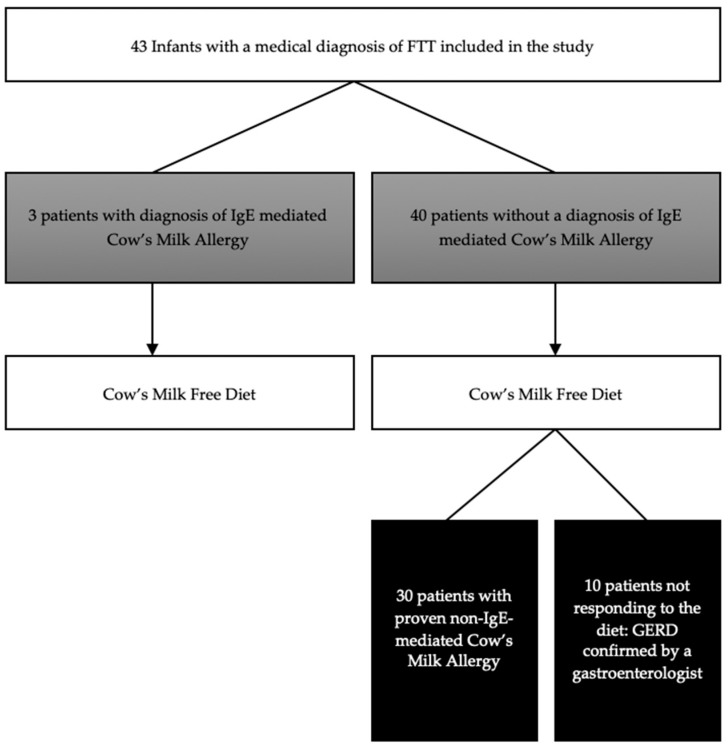
Flow chart showing the diagnosis reached in the 43 included patients (GERD: gastro esophageal reflux disease).

All 43 infants underwent the allergy work-up (SPT and specific IgE assessment for CM) and then started a CMFD (Figure 2) with the same hypoallergenic eHF; 24 of them (56%) were exclusively formula-fed infants. A maternal cow’s milk free diet was started in the remaining 19 (44%) partially breast-fed infants.

In 33 out of 43 infants, FTT was proven to be secondary to a hypersensitivity to cows’ milk (77% of the analyzed group) (Figure 2). In three patients (7%) presenting with symptoms evocative of IgE mediated CMA (immediate urticaria for all the three of them), the allergy work-up (SPT, Specific IgE, and OFC) confirmed the diagnosis of CMA. Median age at OFC was 9 months (SD 4.0). The other 30 patients had a non-IgE-mediated CMA, confirmed by the elimination diet for diagnostic purposes that led to a significant improvement of symptoms and recrudescence after milk re-introduction. In our cohort of 43 infants, FTT was correlated to CMA essentially in infants aged 6 months or less (98% of children, *p* < 0.001). A diagnosis reached before the age of 6 months correlated to an odds ratio (OR) of 15.13, while the OR in patients with more than 6 months of age was only 0.07. The main CMA symptom was digestive, 19 of them (63%) had regurgitation, 6 (20%) had formed stools (Type III/IV Bristol) and 5 (17%) had soft stools (Type V Bristol). In all 30 patients during follow-up, significant improvements were shown in all growth indices within the 3-months of a CMFD independently from the age at which the FTT was manifested (Pr = 0.766). One patient was lost at follow-up (Table 3). Milk tolerance was reached at a median age of 15.8 months (SD 4.1). 

In the 10 patients (23%) not presenting with CMA, digestive symptoms (vomiting) persisted despite eHF trial. For such reason, in this subgroup, we prescribed a thickened eHF with locust bean gum and acid inhibitors. Such approach showed clinical improvements in all 10 children, as confirmed at a follow up evaluation by a specialist in pediatric gastroenterology, who confirmed a diagnosis of GERD (Figure 2). 

## 4. Discussion

Failure to thrive is a descriptive term for insufficient growth, usually identified in infancy. It is a state of inadequate growth rate, if compared with the norms for infants/children of similar age, gender, and ethnicity. Anthropometric indices for assessing FTT are still a matter of debate. Although, no single anthropometric method alone seems adequate to identify weight faltering in the general population [14], age-related weight, assessed over multiple occasions, being also influenced by recent changes in health or nutritional status, remains the simplest and the most reasonable marker of FTT [17].

In the present study, we included 43 infants with a diagnosis of FTT, without a previous diagnosis of chronic medical or surgical conditions. We tried to see if in these patients, CMA was a possible trigger of growth deficiency. In fact, being a multifactorial condition, FTT might also be considered as a wake-up call for underlying allergic disorders. CMA represents the most common food allergy in infancy [18]. It is well known that CMA may lead to FTT. However, the diagnosis of CMA in infants is often complex, especially in those with negative allergy tests and GI-FA disorders [9,11]. 

A practical tool to suspect CMA is represented by the CoMiSS™ scale [12]. It was developed in 2014 by a panel of experts to increase awareness on CMA [12]. The score proposed by the panel includes five different symptoms: Crying, regurgitation, stools modification, skin symptoms, and respiratory symptoms [12]. In case of immediate, IgE mediated hypersensitivity of cow’s milk, the allergy work-up may easily bring to a firm diagnosis: Performing SPT, evaluating specific IgEs, and challenging the patient with the suspected culprit food are the three essential steps to reach a conclusion and prescribe an adapted diet. In our cohort, 7% of infants evaluated for FTT, also presented immediate urticaria following milk exposure. All of them resulted positive to the allergy work-up for cow’s milk, and an elimination diet helped them to resolve both the skin symptoms and the growth retardation. 

It is important therefore, to collect a complete and detailed clinical history to assess the presence of other symptoms, besides FTT, possibly indicating the presence of an immediate hypersensitivity reaction as well. Nevertheless, in those cases in which the history reported by the patient’s caregivers is not immediate, reaching a diagnosis is still quite difficult, and even the suspect of a delayed mechanism of food hypersensitivity may be difficult to evoke. In these cases, the use of the CoMiSS™ may be helpful in targeting those patients who might benefit from an elimination diet. 

An early recognition of non-IgE mediated allergies is indeed essential to set a correct dietary management that may decrease the risk of FTT in both exclusively and partially breast-fed infants. Non-IgE-GI-FA is characterized by sub-acute or chronic gastrointestinal symptoms with usual onset in infancy, and classically include FPE, FPIAP, and food protein-induced enterocolitis syndrome (FPIES) [12]. Celiac disease and CMA–induced iron deficiency anemia are classified as non-IgE-GI-FAs as well [16,17]. Other non-IgE-mediated GI diseases, such as eosinophilic esophagitis, eosinophilic gastritis and eosinophilic gastroenteritis, may induce chronic or intermittent emesis, gastroesophageal reflux (GER) and poor appetite, often leading to FTT in children [11]. Among non-IgE-GI-FA, weight faltering is a cardinal feature of FPE but may often be present in both, acute and chronic-FPIES [19] and in a subset of patients affected by GERD [20,21]. Physiological GER occurs in healthy infants multiple times a day and becomes less common as a baby gets older, while GERD is present when the pathological reflux causes troublesome symptoms (gastro-esophageal and extra-esophageal) and/or pathologic complications such as FTT [22] Younger patients and particularly infants tend to have more extra-intestinal symptoms [23]. It has been demonstrated that isolated gastrointestinal dysmotility may be caused by non-IgE-GI-FA in a subset of patients with gastroesophageal reflux, refractory to optimal medical management [17]. CMA symptoms may, therefore, overlap with GERD [18]. Due to similar presentation, it may be challenging for clinicians to discriminate between primary GERD and food allergy driven GERD symptoms, when considering the clinical picture alone. A double-blind or open challenge showed that 56% of children with severe GERD were suffering from CMA as well [24,25]. Although, a specific immunologic mechanism has not been clearly demonstrated, symptomatic improvement after specific food protein elimination (usually cow milk-based formulas), and recurrence upon re-exposure, supports the role of food hypersensitivity. The basic treatment of GER secondary to FA, or for other types of non-IgE-GI-FA, besides FPIES, is the introduction of elimination diets. In case of patients presenting with chronic FPIES, an oral challenge may be indicated to reach the diagnosis [19]. In our population of infants, all 30 patients with a negative allergy work-up and unspecific non-IgE-GI-FA symptoms showed growth normalization after a CMFD. In 10 patients for which a diet was not sufficient, a diagnosis of GERD was confirmed by a pediatric gastroenterologist and a diet with thickened eHF with locust bean gum and acid inhibitors resolved the growth symptoms. Pediatricians should consider the possibility of gastro-intestinal diseases related to CMA when dealing with infants presenting with FTT. We showed that assessing children presenting with FTT for GERD may be helpful in managing difficult-to-treat conditions.

In recent years, international studies tested the tolerance of eHF and the improvement of growth indices in children with proven CMA [26,27,28]. Therefore, in patients presenting with unspecific symptoms, associated to FTT and possibly to GERD, a CMFD over a period of 4 to 8 weeks may be helpful in highlighting a possible resolution of symptoms. A further re-introduction of milk in the child’s diet may be responsible of the reappearance of symptoms and ultimately confirm the diagnosis of cow’s milk hypersensitivity. In our study, we were indeed able to resolve growth retardation by prescribing eHF, suggesting the presence of un underlying non-IgE-mediated CMA. 

Also, in our study, CMA symptoms mainly appeared during the first months of life, as previously reported [9,29]. Indeed, our data showed that FTT seems to be correlated to CMA essentially in infants aged no more than six months. 

As for the use of the CoMiSS™, Salvatore et al. recently proposed a cut-off of nine to consider the score as positive [30]. The authors highlighted 84% in sensitivity, and 85% specificity for the score, with a 80% positive and 88% negative predictive value. We didn’t have enough data to complete the score for all our patients, but we suggest to add to this score the symptom “FTT” to possibly increase the sensitivity and the positive predictive value of the test. Nevertheless, a prospective study is needed to verify this hypothesis. 

Our study showed some limitations. First, it is a retrospective study conducted in a single center, which could be considered as a selection bias. Also, no biomarker (e.g., serum amino acids, insulin, and blood urea nitrogen) were assessed in the cohort of patients. Nevertheless, the diagnosis of non-IgE-GI-FA is strictly clinical, for the lack of diagnostic tools and biomarkers. To better understand the potential short-term and long-term physiologic benefits of formulas with an evolving nutrition composition, future randomized, double-blind, and controlled studies are needed to compare this new feeding approach with conventional formula-feeding and exclusive breastfeeding. 

Anyway, since there is little literature on this subject focusing on FTT as a clinical aspect of CMA, our data may be considered a modest but significant contribute for early diagnosis especially in non-IgE mediated forms.

In conclusion, even if no symptom or sign alone is specific for the diagnosis of CMA, our findings suggest that infants are more likely to have extra-intestinal symptoms, and those presenting with FTT had a significant response to CMAFD. In this group of patients, feeding modifications (including reduced feeding volumes or more frequent feedings) and the use of eHF or amino-acid based formula may be helpful in resolving the growth problem. The use of eHF is safe and well-tolerated, and showed that formula-fed infants grow in agreement with the WHO growth standards and manifest a healthy early weight gain pattern. We also may speculate that a more sensitive version of the CoMiSS™ should include FTT as a possible manifestation of the disease. Our data show that FTT may be indeed a useful clinical marker for early identification of CMA, particularly in non-IgE mediated forms. 

## Figures and Tables

**Table 1:** Characteristics of the 43 included patients.

General Characteristics		Anthropometrics at First Visit	
Females, *n* (%)	25 (58)	WFA z-score, mean (SD)	−0.7(1.0)
Age in months, mean (SD)	5.7 (2.1)	LFA z-score, mean (SD)	−0.6(1.0)
Gestational age in weeks, mean (SD)	38.7 (1.0)	WFL z-score, mean (SD)	−0.4 (1.1)
Delivery through cesarean section	16 (37)	BMI-for-age z-score, mean (SD)	−0.5 (1.0)
WFA z-score at birth, mean (SD)	−0.1 (1.1)	HCA z-score, mean (SD)	−0.6 (1.0)
LFA z-score at birth, mean (SD)	0.0 (1.3)		
		**Allergy characteristics**	
**Type of feeding at first visit, *n* (%)**		Family history of allergy ‡, *n* (%)	21 (49)
Exclusively formula-fed	24 (56)	Age in months at onset of allergy symptoms, mean (SD)	2.5 (2.3)
Mixed (breastfed/formula-fed)	19 (44)	Skin Prick test positive to other allergens (egg), *n* (%)	2 (5)
**Type of formula used before first visit, *n***		**Types of allergy symptoms, *n* (%)**	
***(duration of use in weeks, mean ± SD)***		Exclusively digestive	40 (93)
Non-hydrolyzed CMP-based formula	43 (8.5 ±5.0)	Digestive and cutaneous	3 (7)

**Legend**—*n*: number of subjects; SD: Standard Deviation; ‡ at least one parent or sibling with confirmed allergy.

**Table 2:** Socio-economic status (SES) of included patients.

PARAMETER		*n* (%)
Place of living		
	Non-rural	37 (86%)
	Rural	6 (14%)
Season of birth		
	Spring	14 (32%)
	Summer	8 (19%)
	Autumn	6 (14%)
	Winter	15 (35%)
Parents’ education		
Mother’s		
	Basic	15 (35%)
	Secondary	18 (42%)
	Higher	10 (23%)
Father’s		
	Basic	16 (37%)
	Secondary	13 (30%)
	Higher	14 (33%)
Pets at home		
	Cat(s)	7 (16%)
	Dog(s)	13 (30%)
	None	23 (54%)
Tobacco smoke exposure		
	At home	17 (40%)
	During pregnancy	13 (30%)

**Table 3:** Growth indices in the 29 patients who completed the follow-up.

Assessed Measurement	At Inclusion	At 90 Days of CMFD	*p*-Value
Weight-for-age z-score, mean (SD)	−0.7 (1.0)	0.1 (0.7)	<0.001 ^a^
Length-for-age z-score, mean (SD)	−0.6 (1.0)	0.0 (1.2)	<0.001 ^a^
Weight-for-length z-score, mean (SD)	−0.4 (1.1)	0.2 (0.6)	<0.001 ^a^
Body mass index-for-age z-score, mean (SD)	−0.5 (1.0)	0.2 (0.7)	<0.001 ^a^
Head circumference-for-age z-score, mean (SD)	−0.6 (1.0)	0.7 (1.0)	<0.001 ^b^

**Legend**—CMFD: Cow’s Milk Free Diet; SD: Standard Deviation; ^a^ Student’s test; ^b^ Wilcoxon’s test.
